# Modulation of Host miRNAs Transcriptome in Lung and Spleen of Peste des Petits Ruminants Virus Infected Sheep and Goats

**DOI:** 10.3389/fmicb.2017.01146

**Published:** 2017-06-26

**Authors:** Aruna Pandey, Amit R. Sahu, Sajad A. Wani, Shikha Saxena, Sonam Kanchan, Vaishali Sah, Kaushal K. Rajak, Alok Khanduri, Aditya P. Sahoo, Ashok K. Tiwari, Bina Mishra, D. Muthuchelvan, Bishnu P. Mishra, Raj K. Singh, Ravi K. Gandham

**Affiliations:** ^1^Computational Biology and Genomics Facility Lab, Division of Veterinary Biotechnology, Indian Council of Agricultural Research-Indian Veterinary Research Institute, BareillyIndia; ^2^Division of Biological Products, Indian Council of Agricultural Research-Indian Veterinary Research Institute, BareillyIndia; ^3^Division of Biological Standardization, Indian Council of Agricultural Research-Indian Veterinary Research Institute, BareillyIndia; ^4^Division of Virology, Indian Council of Agricultural Research-Indian Veterinary Research Institute, NainitalIndia

**Keywords:** microRNAs, PPR, sheep, goats, host–pathogen interaction, molecular pathogenesis

## Abstract

Peste des petits ruminants (PPR) is one of the highly contagious viral disease, characterized by fever, sore mouth, conjunctivitis, gastroenteritis, and pneumonia, primarily affecting sheep and goats. Reports suggested variable host response in goats and sheep and this host response vis-a-vis the expression of microRNAs (miRNAs) has not been investigated. Here, miRNAs were sequenced and proteomics data were generated to identify the role of differentially expressed miRNA (DEmiRNA) in PPR virus (PPRV) infected lung and spleen tissues of sheep and goats. In lungs, 67 and 37 DEmiRNAs have been identified in goats and sheep, respectively. Similarly, in spleen, 50 and 56 DEmiRNAs were identified in goats and sheep, respectively. A total of 20 and 11 miRNAs were found to be common differentially expressed in both the species in PPRV infected spleen and lung, respectively. Six DEmiRNAs—miR-21-3p, miR-1246, miR-27a-5p, miR-760-3p, miR-320a, and miR-363 were selected based on their role in viral infections, apoptosis, and fold change. The target prediction analysis of these six selected DEmiRNAs from the proteome data generated, revealed involvement of more number of genes in lung and spleen of goats than in sheep. On gene ontology analysis of host target genes these DEmiRNAs were found to regulate several immune response signaling pathways. It was observed that the pathways viz. T cell receptor signaling, Rap1 signaling, Toll-like receptor signaling, and B cell receptor signaling governed by DEmiRNAs were more perturbed in goats than in sheep. The data suggests that PPRV-induced miR-21-3p, miR-320a, and miR-363 might act cooperatively to enhance viral pathogenesis in the lung and spleen of sheep by downregulating several immune response genes. The study gives an important insight into the molecular pathogenesis of PPR by identifying that the PPRV—Izatnagar/94 isolate elicits a strong host response in goats than in sheep.

## Introduction

MicroRNAs (miRNAs) are an important part of the host’s regulatory system, involved in post-transcriptional regulation of gene expression in animals, plants, and some DNA viruses (Sevignani et al., 2006). They regulate gene expression by recognizing partial complementary sites, typically within the 3′ untranslated region (3′UTR) of specific mRNAs. Evidence also supports that miRNAs may regulate gene expression by binding to 5′UTR or coding region (Tay et al., 2008; Roberts et al., 2011a). miRNAs are shown to be involved in different biological processes, including reproduction, development, pathogenesis, apoptosis, and signal transduction (Ambros, 2004; Bartel, 2004; Sahu et al., 2015). It has also been suggested that miRNAs may be the effectors in controlling immune regulation, including cellular differentiation and immune response (Rodriguez et al., 2007; Thai et al., 2007; Johnnidis et al., 2008). They are considered as the centered factors in the interaction network between viruses and host. Studies demonstrated that numerous cellular miRNAs (host miRNAs) play a regulatory role in the host–virus interaction network (Scaria et al., 2006; Grassmann and Jeang, 2008).

Cellular miRNAs can greatly influence viral replication and pathogenesis by promoting or inhibiting virus replication (Guo et al., 2013; Li et al., 2014; Mizuguchi et al., 2015). Viral infection also exerts a profound impact on cellular miRNA expression profile, by altering the expression of cellular miRNAs, thereby regulating host or viral RNA targets (Skalsky and Cullen, 2010). It has been observed that miR-142 suppresses replication of Eastern Equine Encephalitis virus (Trobaugh et al., 2014) and miR-122 enhances replication of Hepatitis C virus (Chang et al., 2008). HIV-1, boosts the expression of several host miRNAs, including miR-122, miR-370, miR-373, and miR-297 and suppresses the expression of the miR-17-92 cluster via an unknown mechanism (Roberts et al., 2011b). The emergence of deep sequencing technology has overcome the limitations of miRNA research. Several studies have explored this technology to evaluate global changes in miRNAs expression in response to virus infection (Wang et al., 2009; Cui et al., 2010).

*Peste des petits ruminants* (PPR) is an acute, highly contagious viral disease of sheep and goats characterized by fever, sore mouth, conjunctivitis, gastroenteritis, and pneumonia. Goats have been found to be more susceptible with severe form of clinical disease than sheep (Lefevre and Diallo, 1990; Nanda et al., 1996; Dhar et al., 2002; Singh et al., 2004a; Delil et al., 2012; Truong et al., 2014). It has also been observed that the rate of recovery is lower in goats than in sheep (Singh et al., 2004a). However, severe outbreaks of PPR in regions having large sheep populations have also been reported (Singh et al., 2004a; Raghavendra et al., 2008; Maganga et al., 2013). Recently, host–virus interaction studies in PPR have uncovered transcription factors modulating immune response to Sungri/96 live attenuated vaccine strain and predicted an immune signaling pathway that induces immune response (Manjunath et al., 2015, 2017). However, the host miRNAome in PPR has not been explored till date. In the present study, miRNAs were sequenced and proteomics data were generated to examine the effect of PPR virus (PPRV) on host miRNAs expression vis-a-vis protein expression in lung and spleen tissues of sheep and goats infected with PPR.

## Materials and Methods

### Ethics Statement and Animal Experiment

The vaccine potency testing experiment was carried out at ICAR-Indian Veterinary Research Institute Mukteshwar Campus as per the guidelines of Indian Pharmacopoeia-2014. The study was done after obtaining permission from Indian Veterinary Research Institute Animal Ethics Committee (IVRI-IAEC) under the Committee for the Purpose of Control and Supervision of Experiments on Animals (CPCSEA), India. The protocols were approved vide letter no 387/CPCSEA. Animals (ca. 1 year of age) for the experiment were initially tested to be negative for the presence of PPRV antibody by competitive ELISA (Singh et al., 2004b) and serum neutralization test (SNT; Dhinakar Raj et al., 2000). The animals were also found negative for PPRV antigen in nasal, ocular, buccal, and rectal swabs by sandwich ELISA (Singh et al., 2004c). A highly virulent PPRV (Izatnagar/94 - lineage IV) isolate maintained at PPR Laboratory, Division of Virology, Indian Veterinary Research Institute, Mukteshwar was used as a challenge virus (Sreenivasa et al., 2002). The accession number of this isolate is (KR140086.1; Sahu et al., 2017). Splenic suspension (10%) of virulent virus was inoculated subcutaneously (4 ml suspension, 2 ml each at two different sites). The unvaccinated infected group animals were monitored diurnally for, rectal temperature, any secretion from natural orifices, and feeding habit throughout the experimental period. The unvaccinated animals infected with the PPRV, developed symptoms characteristics of PPRV. The infected animals in which the temperature dropped subnormal were euthanized at 10 days post-infection. As PPRV is epitheliotropic and lymphotropic virus, the tissue samples—lung (epithelial) and spleen (lymphoid) were collected from PPRV infected sheep and goats (*n* = 2 for each of the species). The counterpart healthy tissues (control) were collected from nearby slaughter house from apparently healthy animals that were screened for the absence of PPRV antigen by sandwich ELISA and antibodies by competitive ELISA and SNT.

### Confirmation of PPRV Infection

PPRV infection was confirmed in lung and spleen tissues by, RT-PCR, qRT-PCR, and sandwich ELISA.

### Small RNA Library Construction

Total RNA from each of the collected samples (lung and spleen) was isolated using the RNeasy Mini kit (Qiagen GmbH, Germany) according to the manufacturer’s protocol. The integrity and quantity of isolated RNA were assessed on a Bioanalyzer (Agilent Technologies, Inc). The RNA integrity number (RIN) value of all the samples was found greater than 8, which is considered suitable for further processing (Kukurba and Montgomery, 2015). The library was prepared using NEBNext Multiplex Small RNA Library Prep Kit (New England Biolabs Inc.) following the manufacturer’s protocol. Hundred nanograms of total RNA from each sample was used for small RNA library preparation. The quality of the libraries was assessed on Bioanalyzer. Libraries were quantified using a Qubit 2.0 Fluorometer (Life Technologies) and by quantitative real-time PCR (qPCR; Robin et al., 2016). The high-throughput sequencing was performed on Illumina – NextSeq500 (75 bp single-end) (manufacturer’s protocol).

### miRNAs Prediction and Analysis

The cattle genome sequence was obtained from ftp://ftp.ensembl.org/pub/release-89/fasta/bos_taurus/dna/ The genome was indexed using Bowtie short read aligner program (Langmead et al., 2009). miRNAs are conserved across species (Altuvia et al., 2005). Since there is no complete sheep and goats miRNAs dataset available in miRBase, known mature miRNAs and precursor sequences for cattle were obtained using a Perl script from miRBase database. Data were further processed using miRDeep2 software (Friedlander et al., 2008, 2012). After filtering (read length ≥18 nt) the set of collapsed, non-redundant, clean reads were mapped to the indexed cattle genome using a mapper module. To identify known miRNAs, clean reads were aligned against miRNA precursor sequences reported in the miRBase database using quantifier.pl module. Read counts for each miRNA identified using miRDeep2 were further used for the downstream analysis. The relative expression levels of miRNAs were normalized as TMM (trimmed mean of M-values) using edgeR R/Bioconductor package (Robinson et al., 2010) to identify differentially expressed miRNAs (DEmiRNAs) in lung and spleen.

### Proteomics Data Generation and Data Analysis

#### Protein Extraction and Analysis

Approximately, 1 g of tissue—spleen and lung, from PPRV infected/apparently healthy goats and sheep was taken in 10 ml lysis buffer (50 mM Tris buffer + PIC + PMSF), homogenized on ice, centrifuged at 14,000 rpm and the supernatant was collected into a separate tube (Tris buffer extract). The cell pellet was further added with urea lysis buffer, centrifuged at 14,000 rpm and the resultant supernatant was collected. Supernatants from Tris and urea extractions were run on an SDS-PAGE for quality check (QC) and for further downstream processing. Protein concentration was determined by using Bradford assay and 100 μg of the samples was taken for digestion. Protein samples were treated with 100 mM dithiothreitol at 95°C for 1 h, added with 250 mM iminodiacetic acid and kept at room temperature for 45 min in dark. Samples were then digested with trypsin and incubated overnight at 37°C. Further, 1% of formic acid was added and incubated at 37°C for 45 min. The resulting samples were vacuum dried and dissolved in 10 μl of 0.1% formic acid and centrifuged at 10,000 *g*. The supernatant was injected on C18 Nano-LC column for separation of peptides followed by analysis on the Waters Synapt G2 Q-TOF instrument for MS and MSMS. The raw data was processed by MassLynx 4.1 WATERS. The individual peptides MSMS spectra were matched to the database sequence for protein identification on PLGS software, WATERS. Based on the *m/z* values and their probability to match with a specific peptide present in proteins cleaved at arginine (R) or lysine (K) the protein identification was carried with thresholds, minimum number of peptides to be found for a protein—2; minimum number of fragments (MSMS) ions in a peptide—3; minimum number of fragments (MSMS) ions in a protein—7; peptide mass tolerance—30 ppm; and fragment ion mass tolerance—70 ppm. The identified proteins in the three runs of each sample were compared with each other as control (healthy) and infected samples. Expression Analysis package of the PLGS software was then used for quantification. The ion counts matching with the peptides of a specific protein corresponding between the two samples in the three runs, were averaged and the ratio was calculated for the whole protein.

### Target Prediction of miRNAs

To better understand the biological function of DEmiRNA, TargetScan tool (Aggarwal et al., 2015) with default parameters was used to predict target genes of the selected DEmiRNAs (six miRNAs selected based on their role). From these predicted genes, the dysregulated genes from the proteomics data were identified for the miRNA selected (downregulated proteins for upregulated miRNA and upregulated proteins for downregulated miRNA). These common target genes from TargetScan and proteomics data were considered for further analysis. The miRNA–protein network was created based on the expression profile of target genes and miRNAs using Cytoscape (ver. 3.1.1; Shannon et al., 2003).

### Gene Ontology Enrichment and Pathway Analysis

Functional annotation of the selected DEmiRNAs in each tissue was performed using target genes governed by them in ClueGO (ver. 2.1.4; Bindea et al., 2009) in Cytoscape (ver. 3.1.1; Shannon et al., 2003). Immune system processes and KEGG pathways were selected to generate a functionally organized GO/pathway term networks.

### Validation Using qPCR

Total RNA, including small RNA from the lung and spleen of control and infected sheep and goats were isolated using mirVana^TM^ miRNA isolation kit (Invitrogen). Reverse transcriptase reactions were performed using RT specific primers of miR-363, miR-760-3p, miR-21-3p, and U6snRNA by TaqMan^®^ MicroRNA Reverse Transcription Kit. Real-time PCR was performed using a standard TaqMan PCR kit protocol on an Applied Biosystems 7500 fast Sequence Detection System. The 10 μl PCR included 5 μl of 2× TaqMan Gene Expression Master Mix (Thermo Fisher Scientific Inc., Wilmington, DE, United States, Cat. No. 4369016), 0.5 μl of 20× TaqMan probe, 2 μl (0.134 ng) of RT product and 2.5 μl of NFW. The reactions were incubated in a 96-well plate at 95°C for 10 min, followed by 40 cycles of 95°C for 15 s and 60°C for 1 min. All reactions were run in triplicate. The threshold cycle (Aad et al., 2015) is defined as the fractional cycle number at which the fluorescence passes the fixed threshold. The expression of the selected miRNAs described above was assayed taking the expression of U6snRNA as an internal control. The relative expression of each miRNA was calculated using the 2^-ΔΔCT^ method with a control group as calibrator (Schmittgen and Livak, 2008).

## Results

### Confirmation of PPRV Infection

Viral infection in the lung and spleen of sheep and goats infected with PPRV was confirmed by RT-PCR of 351 bp N gene amplicon in lung and spleen (**Supplementary Figure S1**). The viral infection was further confirmed by sandwich ELISA and qRT-PCR in both lung and spleen of goats and sheep (data not shown).

### miRNAs Prediction and Identification of DEmiRNAs

In goats, miRDeep2 identified 298 and 283 miRNAs in control and PPRV infected lung, and 277 and 274 miRNAs in control and PPRV infected spleen. In sheep, 290 and 298 miRNAs in control and PPRV infected lung, and 274 and 256 miRNAs in control and PPRV infected spleen, respectively, were predicted. DEmiRNAs [FDR ≤ 0.05 and fold change (log_2_FC) ≥ 1] in lung and spleen of PPRV infected sheep and goats are presented in **Table 1**. A total of 67 miRNAs (34 downregulated and 33 upregulated) were dysregulated in the lungs of PPRV infected goats. However, a relatively small number of DEmiRNAs—37 miRNAs (16 miRNAs downregulated and 21 miRNAs upregulated) were identified in the lungs of sheep (**Table 1**). In infected goat’s spleen, 50 miRNAs were dysregulated with 26 of them downregulated and 24 upregulated. In spleen of sheep, 56 miRNAs were differentially expressed after PPRV infection and of these, 26 miRNAs were downregulated and 30 miRNAs were upregulated (**Table 1**).

**Table 1:** Differentially expressed (log_2_FC ≥ 1 and FDR ≤ 0.05 up/down regulated miRNAs in goats and sheep spleen and lung.

Sample	Differentially expressed with log_2_FC > 1 and FDR ≤ 0.05	Down-regulated	Up-regulated	Common DEmiRNAs
Sheep lung	37	16	21	11
Goats lung	67	34	33	
Sheep spleen	56	26	30	20
Goats spleen	50	26	24	

**FIGURE 1:**
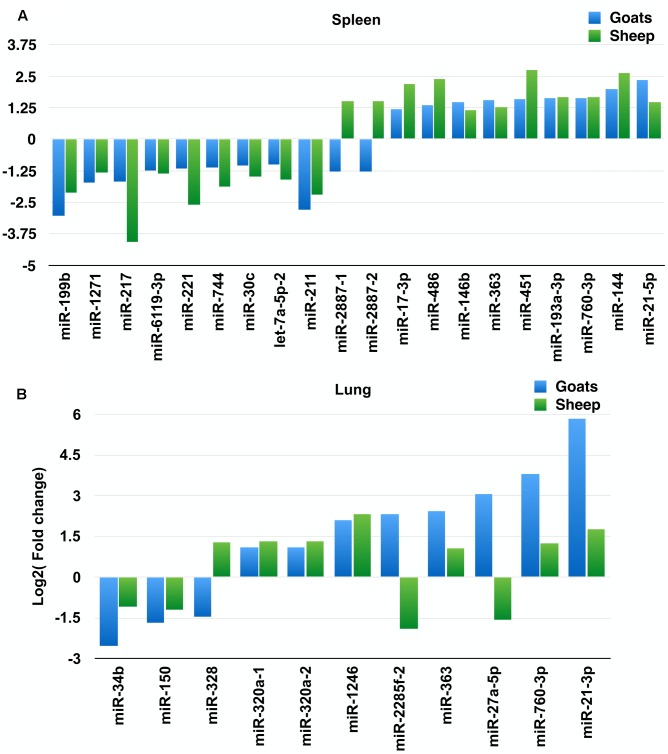
Commonly differentially expressed miRNAs (log_2_FC > 1). **(A)** Commonly differentially expressed miRNAs (log_2_FC > 1) in spleen tissue of sheep and goats. **(B)** Commonly differentially expressed miRNAs (log_2_FC > 1) in lung tissue of sheep and goats.

On comparing tissues across species, 20 and 11 miRNAs were found to be commonly differentially expressed in PPRV infected spleen and lung, respectively, in both sheep and goats. Among these 20 common DEmiRNAs in spleen, 11 DEmiRNAs (miR-199b, miR-1271, miR-217, miR-2887-1, miR-2887-2, miR-6119-3p, miR-221, miR-744, miR-30c, let-7a-5p-2, and miR-211) were downregulated and nine DEmiRNAs (miR-17-3p, miR-486, miR-146b, miR-363, miR-451, miR-193a-3p, miR-760-3p, miR-144, and miR-21-5p) were upregulated in goats, and in sheep, nine DEmiRNAs (miR-199b, miR-1271, miR-217, miR-6119-3p, miR-221, miR-744, miR-30c, let-7a-5p-2, and miR-211) were downregulated and 11 DEmiRNAs (miR-2887-1, miR-2887-2, miR-17-3p, miR-486, miR-146b, miR-363, miR-451, miR-193a-3p, miR-760-3p, miR-144, and miR-21-5p) were upregulated (**Figure 1A** and **Table 2**). Of these 20 common DEmiRNAs, miR-21-5p was the most upregulated (log_2_FC = 2.35) and miR-199b was the most downregulated DEmiRNA (log_2_FC = -3.03) in the spleen of goats. While in infected spleen of sheep the most upregulated DEmiRNAs were miR-451 (log_2_FC = 2.75) and miR-144 (log_2_FC = 2.63) and the most downregulated DEmiRNAs were miR-217 (log_2_FC = -4.08) and miR-221 (log_2_FC = -2.60).

**Table 2:** DEmiRNAs commonly identified in PPRV infected spleen tissue of sheep and goats.

S. no.	miRNAs	Log_2_FC (goats)	Log_2_FC (sheep)	Downregulation/upregulation
1	miR-199b	-3.03	-2.13	Down
2	miR-1271	-1.74	-1.32	Down
3	miR-217	-1.67	-4.08	Down
4	miR-2887-1	-1.29	1.51	Down (goats) Up (sheep)
5	miR-2887-2	-1.29	1.51	Down (goats) Up (sheep)
6	miR-6119-3p	-1.26	-1.36	Down
7	miR-221	-1.15	-2.60	Down
8	miR-744	-1.10	-1.87	Down
9	miR-30c	-1.06	-1.47	Down
10	let-7a-5p-2	-1.00	-1.62	Down
11	miR-211	-2.79	-2.19	Down
12	miR-17-3p	1.168	2.20	Up
13	miR-486	1.32	2.39	Up
14	miR-146b	1.46	1.15	Up
15	miR-363	1.55	1.28	Up
16	miR-451	1.57	2.75	Up
17	miR-193a-3p	1.63	1.67	Up
18	miR-760-3p	1.63	1.67	Up
19	miR-144	1.98	2.63	Up
20	miR-21-5p	2.35	1.44	Up

The expression profile of the 11 common DEmiRNAs in the lung tissue varied in sheep and goats. miR-328 was found downregulated in goats but upregulated in sheep; two miRNAs—miR-2285f-2 and miR-27a-5p were found upregulated in goats but downregulated in sheep; six miRNAs—miR-320a-1, miR-320a-2, miR-1246, miR-363, miR-760-3p, and miR-21-3p were upregulated and two miRNAs—miR-34b and miR-150 were downregulated in both species (**Figure 1B** and **Table 3**). Among these 11 common DEmiRNAs, the expression of miR-21-3p, miR-760-3p, and miR-27a-5p was more abundant in lungs of PPRV infected goats with log_2_ fold change of 5.82, 3.79, and 3.07, respectively, while miR-34b (log_2_FC = -2.53) and miR-2285f-2 (log_2_FC = -2.53) were found least abundant in lungs of goats and sheep, respectively.

**Table 3:** DEmiRNAs commonly identified in PPRV infected lung tissue of sheep and goats.

S. no.	miRNAs	Log_2_FC (goats)	Log_2_FC (sheep)	Downregulation/upregulation
1	miR-34b	-2.53	-1.07	Down
2	miR-150	-1.68	-1.20	Down
3	miR-328	-1.46	1.29	Up (goats) Down (sheep)
4	miR-320a-1	1.08	1.30	Up
5	miR-320a-2	1.08	1.30	Up
6	miR-1246	2.10	2.31	Up
7	miR-2285f-2	2.34	-1.89	Up (goats) Down (sheep)
8	miR-363	2.42	1.04	Up
9	miR-27a-5p	3.07	-1.57	Up (goats) Down (sheep)
10	miR-760-3p	3.79	1.24	Up
11	miR-21-3p	5.82	1.75	Up

**FIGURE 2:**
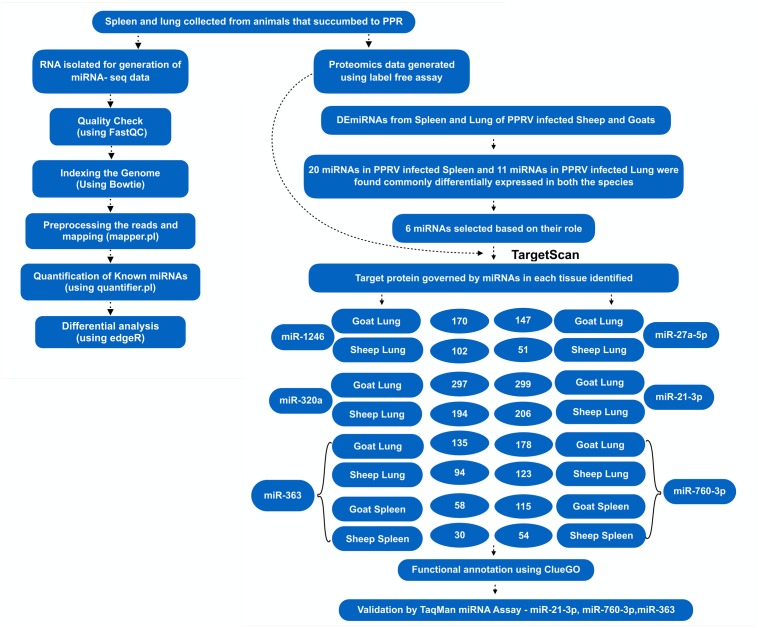
Overview of the miRNA prediction and identification of miRNAs that regulated dysregulated proteins.

Among these 31 commonly DEmiRNAs, six DEmiRNAs were selected based on their role in viral infections, apoptosis, and fold change (**Table 4**). These miRNAs include miR-21-3p, miR-320a, miR-27a-5p, and miR-1246—expressed in lung of both species; miR-760-3p and miR-363—expressed in lung and spleen of both species (**Figure 2**). The miRNAs—miR-363 and miR-760-3p commonly present in PPRV infected lung and spleen of both species were identified to be upregulated. In lung, the expression of miR-363 and miR-760-3p was higher in goats with log_2_ fold change of 2.42 and 3.79, respectively, than in sheep (miR-363, log_2_FC = 1.04 and miR-760-3p, log_2_FC = 1.24) after PPRV infection. In spleen, the expression profile of miR-363 was higher in goats (log_2_FC = 1.55) than in sheep (log_2_FC = 1.28), however, no difference in expression of miR-760-3p was observed in goats (log_2_FC = 1.63) and sheep (log_2_FC = 1.67) infected with PPRV.

**Table 4:** Six selected DEmiRNAs.

miRNAs	Role	Tissue	Reference
miR-27a-5p	Repression of viral replication	Lung	Roberts et al., 2011b
miR-21-3p	Induce apoptosis	Lung	Lo et al., 2013
miR-320a	Inhibit virus infection	Lung	Sun et al., 2014
miR-1246	Promotes virus cytotoxicity	Lung	Sheng et al., 2014
miR-363	Induced apoptosis	Lung and spleen	Zhang et al., 2014
miR-760-3p	Very highly upregulated	Lung and spleen	–

### Target Prediction and miRNA–Protein Regulatory Network Analysis

**FIGURE 3:**
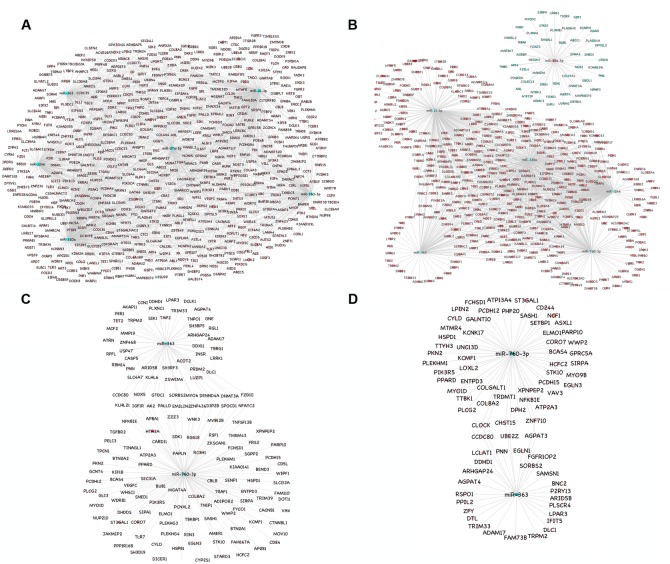
miRNA-mRNA regulatory network. **(A)** Network of upregulated miRNAs (miR-21-3p, miR-27a-5p, miR-1246, miR-320a, miR-363, and miR-760-3p) and their downregulated genes in the lung tissue of goats. **(B)** Network of upregulated miRNAs (miR-21-3p, miR-1246, miR-320a, miR-363, and miR-760-3p) and their downregulated genes and the downregulated miRNA (miR-27a-5p) and its upregulated genes in the lung tissue of sheep. **(C)** Network of upregulated miRNAs (miR-363 and miR-760-3p) and their downregulated genes in the spleen tissue of goats. **(D)** Network of upregulated miRNAs (miR-363 and miR-760-3p) and their downregulated genes in the spleen tissue of sheep. Blue nodes indicate upregulation and red nodes indicates downregulation. Color intensity denoted the level of gene expression.

A total of 1149 (714 downregulated, 435 upregulated) and 1565 (1041 downregulated, 524 upregulated) differentially expressed proteins were identified in lung of sheep and goats, respectively, and 944 (281 downregulated, 663 upregulated) and 909 (590 downregulated, 319 upregulated) differentially expressed proteins were identified in spleen of sheep and goats, respectively. The number of dysregulated proteins identified through mass spectrophotometry by each of these six miRNAs is shown in **Figure 2**. The miRNA–protein interactions for each species and tissue are represented in a network (**Figure 3**). In the miRNA–protein network of lung tissue of goats, three miRNAs—miR-21-3p, miR-363, and miR-320a mutually regulate EGFR (epidermal growth factor receptor), which is involved in immune response. Similarly, IGF1R (insulin like growth factor 1) protein, involved in regulation of immune response was the target of miR-27a-5p, miR-363, miR-320a, and miR-760-3p. The TRIM (tripartite motif family) family members TRIM24, TRIM36, and TRIM45 were identified to be modulated by miR-1246, miR-320a, and miR-21-3p, respectively. The expression level of NF-κB signaling-related molecules IRAK2 and TRAF4 was regulated by miR-1246 and miR-320; and miR-1246 and miR-760-3p, respectively (**Figure 3A**). In PPRV infected sheep lung, the upregulated miRNAs—miR-21-3p and miR-320a govern immune genes—TRAF6, EGFR, and ERBB4 and the downregulated miR-27a-5p potentially modulates the expression of genes—MAP3K7 and MAPK8IP3, involved in JNK signaling pathways (**Figure 3B**).

In PPRV infected spleen of goats, miR-363 and miR-760-3p regulate apoptotic molecules (NFATC2, NOX5, and MYO6) and target genes involved in immunological processes (NFATC2, IGF1R, KLHL21, and NOX5) (**Figure 3C**). The immune effector molecules IFIT5 and TRIM33 were targeted by miR-363, and CD244 and NFKBIE were regulated by miR-760-3p in infected spleen of sheep (**Figure 3D**).

### Gene Ontology and KEGG Pathway-Based Network Analysis

**FIGURE 4:**
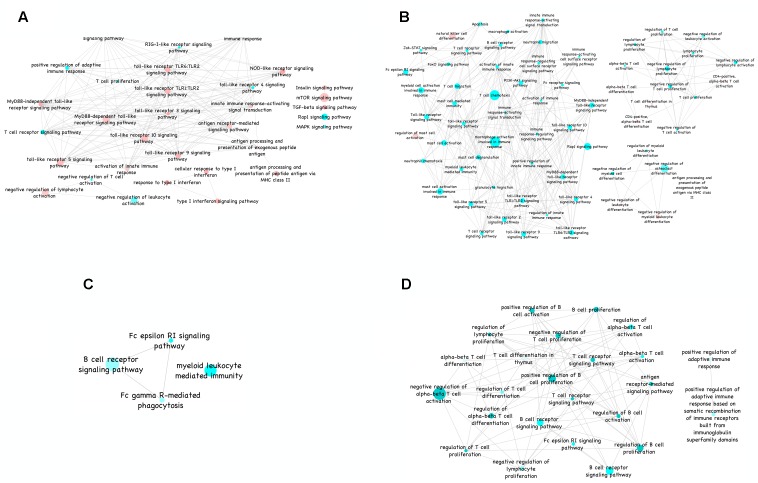
Gene ontology of immune-related KEGG pathways. **(A)** Gene ontology of immune-related KEGG pathways in sheep lung visualized in ClueGO (ver. 2.1.4) plugin of Cytoscape (ver. 3.1.1). **(B)** Gene ontology of immune-related KEGG pathways in goats lung visualized in ClueGO (ver. 2.1.4) plugin of Cytoscape (ver. 3.1.1). **(C)** Gene ontology of immune-related KEGG pathways in sheep spleen visualized in ClueGO (ver. 2.1.4) plugin of Cytoscape (ver. 3.1.1). **(D)** Gene ontology of immune-related KEGG pathways in goats spleen visualized in ClueGO (ver. 2.1.4) plugin of Cytoscape (ver. 3.1.1). Color from red to green of the nodes in the network depicts increase in significance. The diameter indicates the percent associated genes to a particular node.

Functional annotation of a total of 770 and 1226 target proteins governed by selected six miRNAs in infected sheep and goats infected lung, respectively, resulted in higher number of significantly enriched pathways and GO terms in goats than in sheep (**Figure 4**). The highly enriched common GO terms and pathways targeted by the miRNAs in the lung tissue of sheep and goats includes T cell receptor signaling pathway, Rap1 signaling pathway, Toll-like receptor TLR6:TLR2 signaling pathway, etc. (**Figures 4A,B**).

Similarly, functional annotation of 84 and 173 target proteins governed by two out of selected six miRNAs in infected sheep and goats spleen, respectively, identified enrichment of B cell receptor signaling and FC epsilon RI signaling pathways in both the species (**Figures 4C,D**). Furthermore, targets of DEmiRNAs in sheep spleen were also found enriched in FC-gamma R mediated phagocytosis and myeloid leukocyte mediated immunity. The targets of DEmiRNAs of goat’s spleen were found involved in NF-κB signaling pathway, alpha–beta T cell differentiation, ErbB signaling pathway and regulation of B cell activation. In addition, the number of significantly enriched pathways and GO terms were higher in lung and spleen tissue of goats than in sheep.

### Validation of DEmiRNAs by qPCR

**FIGURE 5:**
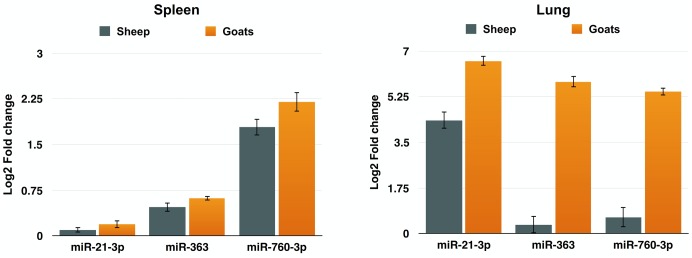
qPCR validation of sequencing data. The change in the miRNA expression of miR-21-3p, miR-363, and miR-760-3p was calculated with U6snRNA as reference gene for normalization. Fold change is represented as log_2_FC.

To further validate the expression of DEmiRNAs from high-throughput sequencing, qPCR was performed on three DEmiRNAs—miR-21-3p, miR-363, and miR-760-3p. The expression of miR-363 and miR-760-3p in sheep and goats in both the tissues was in concordance with small RNA sequencing results. The expression of miR-21-3p was found to be in concordance with the sequencing results in PPRV infected spleen of both the species. However, in infected lung miR-21-3p was found upregulated on qPCR though not found in small RNA sequencing data in both the species (**Figure 5** and **Table 5**).

**Table 5:** qPCR validation of small RNA sequencing data.

	Lung (goats)		Lung (sheep)		Spleen (goats)		Spleen (sheep)	
miRNAs	qPCR (log_2_ fold change	miRNA-seq (log_2_ fold change)	qPCR (log_2_ fold change)	miRNA-seq (log_2_ fold change)	qPCR (log_2_ fold change)	miRNA-seq (log_2_ fold change)	qPCR (log_2_ fold change)	miRNA-seq (log_2_ fold change)
miR-21-3p	+6.619	+5.826	+4.344	+1.753	+0.189	–	+0.097	–
miR-363	+5.823	+2.425	+0.345	+1.049	+0.618	+1.555	+0.474	+1.286
miR-760-3p	+5.433	+3.798	+0.629	+1.242	+2.199	+1.637	+1.784	+1.679

## Discussion

PPR is a major threat to livestock keepers in developing countries, causing a severe disease in goats and sheep. Host encoded miRNAs have been demonstrated to be key regulators of host–virus interactions, and their expression is often affected by viral infection (Hussain and Asgari, 2010; Skalsky and Cullen, 2010). Currently there is no report available suggesting PPRV infection-induced changes in expression of cellular or host miRNAs. In the present study, we investigated the expression pattern of host miRNAs in spleen and lung of sheep and goats infected with PPRV vis-a-vis protein expression.

Detailed analysis revealed many differences in the global expression profile of miRNAs among lung and spleen, suggesting common and unique miRNA transcriptome landscape against PPRV. PPRV infection altered the expression of host miRNAs in lung and spleen. Under PPRV infection, a total of 37 and 67 DEmiRNAs were identified in lung of sheep and goats; and, 56 and 50 DEmiRNAs in the spleen of sheep and goats, respectively. PPRV infection in spleen and lung triggered the expression of many immune-related miRNAs, including, miR-21, miR-150, miR-146b, and let-7 family as reported in Japanese encephalitis virus infection (Cai et al., 2015). Moreover, 20 and 11 common DEmiRNAs expressed in spleen and lung of both species, respectively, suggested variable tissue response to PPRV infection. Among these 31 DEmiRNAs, six DEmiRNAs—miR-21-3p, miR-320a, miR-27a-5p, miR-1246 (expressed in lung of both species), miR-760-3p and miR-363 (expressed in lung and spleen of both species) were selected based on their role in viral infection, apoptosis and fold change. In infected goat’s lung all these six DEmiRNAs were found to upregulated. However, in infected sheep’s lung, miR-27a-5p was found to be downregulated and the rest of the five DEmiRNAs were upregulated.

miR-21-3p induce apoptosis (Lo et al., 2013) and PPRV is also reported to cause apoptosis of host cells (Mondal et al., 2001). The upregulated miR-363 is also known to induce apoptosis (Zhang et al., 2014; Zhou et al., 2014; Hu et al., 2015; Li et al., 2015). The upregulation of miR-21-3p and miR-363 in PPRV infections suggests synergistic effect of these miRs along with the virus in inducing apoptosis. The upregulation of miR-363 has also been reported in Human papillomavirus (HPV)+ and HPV- pharyngeal squamous cell carcinoma and in HPV16+ HNSCC cell lines (Lajer et al., 2011; Wald et al., 2011). Recently, miR-27a-5p was found to be highly expressed in vaccinia virus infection (Buck et al., 2010). Under PPRV infection miR-27a-5p was found to be upregulated in infected lung of goats but downregulated in sheep suggesting a species-specific response. Further, miR-320a is known to inhibit mink enteritis virus infection by downregulating its receptor, transferrin receptor (TfR; Sun et al., 2014). Significant upregulation of miR-320a expression in PPRV infected lung tissue of sheep and goats suggests that miR-320a might serve in triggering antiviral response against PPRV infection. Sheng et al. (2014) reported that the upregulated miR-1246 decreased the expression of cell adhesion target genes and hence promotes the cytotoxicity induced by Ebola virus glycoprotein. Similarly, upregulated miR-1246 was found to promote cell death pathway by reducing the expression levels of DLG3 protein during HEV71 infection in human neuroblastoma cells (Xu et al., 2014). The increased expression of miR-1246 in the PPRV infected lung of goats and sheep in our study, could be a factor contributing to the pathogenesis of PPRV.

For miRNA studies, it is critical to identify targets for understanding its biological function and molecular mechanism (Tang et al., 2015). The miRNA–protein network analysis suggests that one miRNA could participate in several biological processes by targeting different mRNAs, and one biological process could be influenced by multiple miRNAs. The upregulated miR-27a-5p, miR-363, miR-320a, and miR-760-3p were observed to bring about the downregulation of IGF1R in the PPRV infected lung of goats. IGF1R, is a multifunctional receptor that plays an important role in the regulation of immune response, including cell differentiation and proliferation (Smith, 2010). Similarly, TRIM24, tripartite motif containing 24, which is involved in cytokine signaling and secretion (Tisserand et al., 2011) was downregulated by miR-1246 and miR-320a in lung tissue of goats. miRNAs play an important role in regulation of NF-κB signaling pathway during viral infections (Gao et al., 2014) and activation of NF-κB is important for immune defense (Hoesel and Schmid, 2013). NF-κB signaling-related molecules IRAK2 were found modulated by miR-1246 and miR-320a, and TRAF4 was found modulated by miR-1246 and miR-760-3p in PPRV infected lung of goats in the present study. Interferon (IFN)-mediated pathway is a crucial part of the cellular response against viral infection (Wu et al., 2015). TRAF6, a major element in IFN production (Yoshida et al., 2008) was suppressed by PPRV-induced miR-21-3p and miR-320a in the lung of sheep. Similarly, the expression of IFIT5, which is involved in stimulating anti-viral response (Zhang et al., 2013) was suppressed by miR-363 in the spleen of sheep. This suggests that PPRV-induced miR-21-3p, miR-320a, and miR-363 might act cooperatively to enhance viral pathogenesis in the lung and spleen of sheep by downregulating several immune response genes. Further, this could be corroborated by the GO and pathway analysis of the potential targets of all the six DEmiRNAs. It was observed that the pathways governed by DEmiRNAs were more perturbed in goats than in sheep, thereby reflecting on the severity of disease in goats than in sheep.

## Conclusion

This study demonstrated for the first time DEmiRNAs in sheep and goats under PPRV infection. The DEmiRNAs identified in this study govern genes involved in immune response processes. It was observed that PPRV elicits a strong host response in goats than in sheep as evident from the number of significantly enriched immune system pathways and genes perturbed. This study revealed that PPRV-induced miR-21-3p, miR-320a, and miR-363 might act cooperatively to enhance viral pathogenesis, which warrants further research.

## Author Contributions

RS, BPM, AT, and RG conceived and designed the research. ARS, SW, SS, SK, and AK conducted the wet lab work. AP, SW, ARS, and RG analyzed the data. AP, ARS, SW, and RG wrote the manuscript. AP, ARS, SW, RG, VS, APS, KR, BM, and DM helped in manuscript drafting and editing. RS, BPM, AT, and RG proofread the manuscript.

## Conflict of Interest Statement

The authors declare that the research was conducted in the absence of any commercial or financial relationships that could be construed as a potential conflict of interest.

## Supplementary Material

Figure S1 Confirmation of PPRV infection in spleen and lung tissues of sheep and goats. Amplification of 351 bp N gene by RT-PCR. Lane M, 100 bp ladder; lane 1, NTC; lane 2, sheep spleen; lane 3, goats spleen; lane 4, goats lung; lane 5, sheep lung; lane 6, PPRV positive control.
