# Supplementary figures and images for: Modulation of Host miRNAs Transcriptome in Lung and Spleen of Peste des Petits Ruminants Virus Infected Sheep and Goats
Source: Front Microbiol. 2017 Jun 26;8:1146. doi: 10.3389/fmicb.2017.01146 (PMC5483481; doi:10.3389/fmicb.2017.01146)

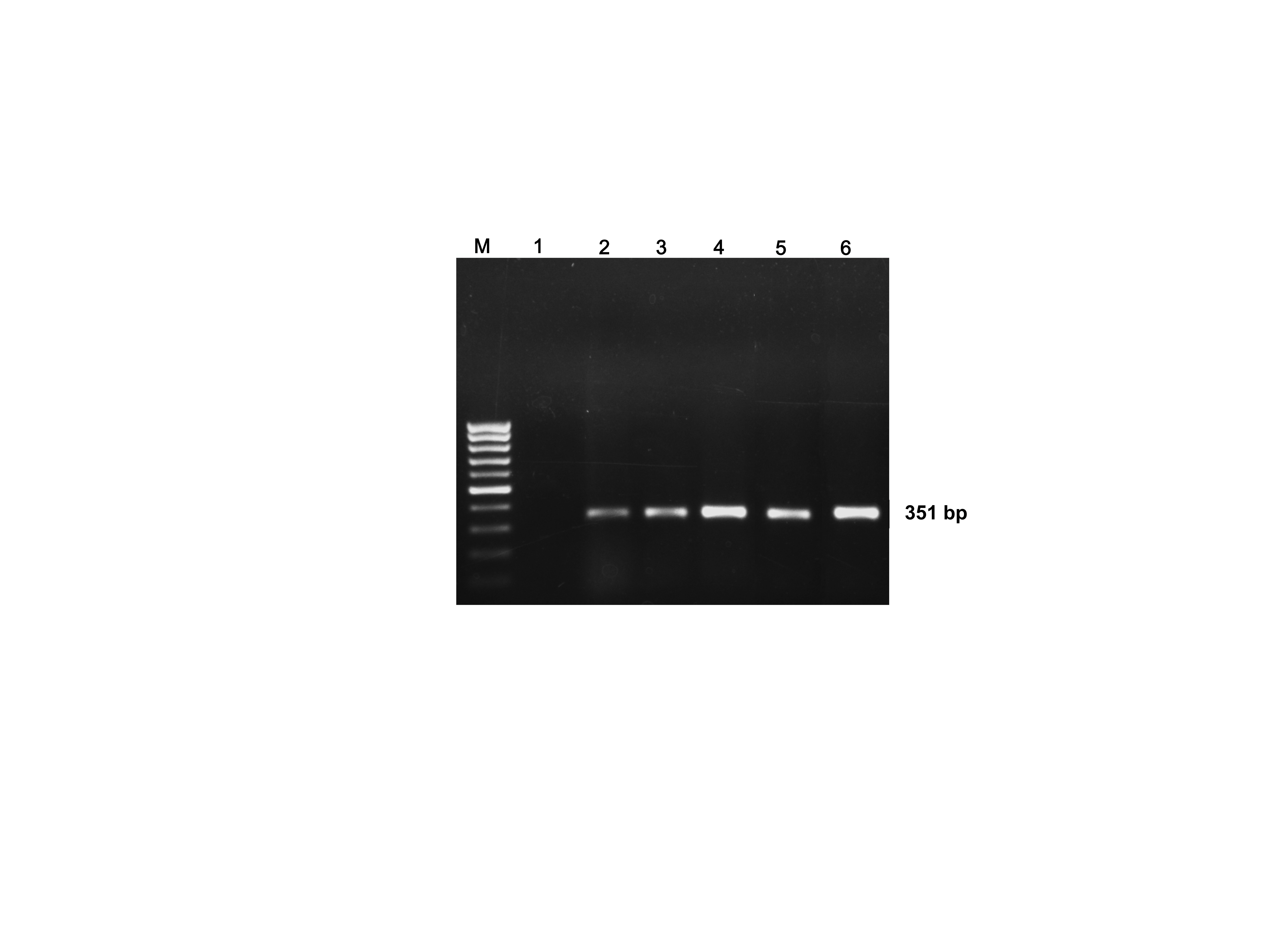

Supplement: Figure S1 — Confirmation of PPRV infection in spleen and lung tissues of sheep and goats. Amplification of 351 bp N gene by RT-PCR. Lane M, 100 bp ladder; lane 1, NTC; lane 2, sheep spleen; lane 3, goats spleen; lane 4, goats lung; lane 5, sheep lung; lane 6, PPRV positive control. [file Image_1.JPEG]
